# The Tromso Infant Faces Database (TIF): Development, Validation and Application to Assess Parenting Experience on Clarity and Intensity Ratings

**DOI:** 10.3389/fpsyg.2017.00409

**Published:** 2017-03-24

**Authors:** Jana K. Maack, Agnes Bohne, Dag Nordahl, Lina Livsdatter, Åsne A.W. Lindahl, Morten Øvervoll, Catharina E. A. Wang, Gerit Pfuhl

**Affiliations:** Department of Psychology, UiT – The Arctic University of NorwayTromsø, Norway

**Keywords:** face processing, infant emotional expressions, validation study, baby schema, parent-infant attachment, non-verbal communication

## Abstract

Newborns and infants are highly depending on successfully communicating their needs; e.g., through crying and facial expressions. Although there is a growing interest in the mechanisms of and possible influences on the recognition of facial expressions in infants, heretofore there exists no validated database of emotional infant faces. In the present article we introduce a standardized and freely available face database containing Caucasian infant face images from 18 infants 4 to 12 months old. The development and validation of the Tromsø Infant Faces (TIF) database is presented in Study 1. Over 700 adults categorized the photographs by seven emotion categories (happy, sad, disgusted, angry, afraid, surprised, neutral) and rated intensity, clarity and their valance. In order to examine the relevance of TIF, we then present its first application in Study 2, investigating differences in emotion recognition across different stages of parenthood. We found a small gender effect in terms of women giving higher intensity and clarity ratings than men. Moreover, parents of young children rated the images as clearer than all the other groups, and parents rated “neutral” expressions as more clearly and more intense. Our results suggest that caretaking experience provides an implicit advantage in the processing of emotional expressions in infant faces, especially for the more difficult, ambiguous expressions.

## Introduction

Successful social interaction depends on the ability to perceive and understand emotional expressions. Face processing is crucial in this regard, as faces carry valuable information about attentional focus, physical health and identity such as age, ethnicity and gender, as well as about emotions, pain and pleasure (Jack and Schyns, 2015). Indeed, “reading a face” is often effortless and fast despite faces being rich on information and high-dimensional. This effortless processing of such complex information is due to predispositional, nearly immediate, learning about faces. That is, within hours after birth infants show preference for faces (Johnson et al., 1991; Walker-Andrews, 1997), indicating the evolutionary importance of face processing. The infant detects facial information that eventually will enable recognition and discrimination of emotion (Walker-Andrews, 1997). Furthermore, for newborn babies and infants who are highly dependent on adult care, crying and facial expressions are the main means of communicating their physiological and emotional states and needs (Sullivan, 2014). Accordingly, humans of all ages should be able to read these signals. Indeed, adults’ attention to and attunement with infant emotional expressions promotes healthy infantile development and is essential for human offspring survival (Fonagy and Target, 1997; Stern, 2002). Lorenz (1943, 1971) was the first to propose that infants have specific physical features that attract attention and enhance caretaking behavior in adults, known as the “kindchenschema” or baby schema. There is indeed strong evidence suggesting that infant stimuli are prioritized in the attentional system of adults (Brosch et al., 2007; Parsons et al., 2011; Hahn et al., 2013; Borgi et al., 2014), especially if they display emotional content (Phelps et al., 2006; Brosch et al., 2007; Thompson-Booth et al., 2014a).

To study the social importance of facial expressions, as well as identifying the mechanisms and functions of emotions, photographs of faces with different emotional expressions have been commonly used. However, heretofore there is no validated database of infant facial expressions. Therefore, in the present article we introduce a standardized face database containing Caucasian infant face images from 18 infants. The development and validation of the Tromsø Infant Faces (TIF) database is presented in Study 1. In order to examine the relevance of TIF, we then present its first application in Study 2, investigating differences in the emotion recognition accuracy, as well as in the clarity, intensity and valence ratings when comparing men and women at different stages of parenting.

## Study 1: Development and Validation of the TromsØ Infant Faces (TIF) Database

Standardized databases for facial expressions have been developed, including for example: the “Pictures of Facial Affect” (POFA) database (Pictures of Facial Affect [POFA], 2015), the “Karolinska directed emotional faces” (KDEF) database (Lundqvist et al., 1998) and the Radboud faces database (Langner et al., 2010). The POFA uses adults of all ages whereas the KDEF uses young adults (students). The Radboud database includes both adults and children, but not under the age of 6 years. The child affective face set (CAFE) was newly developed to allow for research on perception of children’s affective facial expressions (Lobue and Thrasher, 2015). CAFE contains photographs of children aged 2–8 years displaying seven different facial expressions – happy, angry, sad, fearful, surprise, disgust, and neutral. These are the six basic emotions described by Ekman and Cordaro (2011) plus a neutral facial expression. Neither of the databases includes facial expressions of infants. More general databases of emotional stimuli can include infant images but those photos are not standardized for facial expressions, and include often only laughing babies, e.g., GAPED (Dan-Glauser and Scherer, 2011), the *“*International Affective Picture System” (IAPS) (Lang et al., 2008) or the “Emotional Picture Set” (EmoPicS) (Wessa et al., 2010).

During infancy, the characteristics of the infant’s face and facial expressions change considerably. Due to fat tissue and facial muscles, a newborn’s emotional expression is not as clearly recognizable as that of a toddler’s (Camras and Shutter, 2010). That is, as the baby schema lessens, the expression becomes clearer. The baby schema is found to be strongest before the age of 1 year (Lorenz, 1943, 1971; Hildebrandt and Fitzgerald, 1979; Glocker et al., 2009), indicating a notable development in the facial features during the infant’s first year of life. Moreover, facial expressions are found to be intimately linked to cognitive development (Lewis and Michalson, 1983). The brain develops extensively during infancy and there is evidence suggesting that while facial expressions initially are organized subcortically, over time they get integrated into higher cognitive and emotional systems (Sullivan and Lewis, 2003). Accordingly, while newborns display most components of the human expression repertoire, their expressions are still brief and subtle (Lewis and Michalson, 1983; Sullivan and Lewis, 2003). Often the expressions are not pure, but rather blending together several emotion signals (Sullivan and Lewis, 2003). For example, the combination “anger”/”sad” is commonly seen in infants. Even in adults, discrete, basic emotions are best understood as families or groups of related states each having their characteristic facial and vocal features, autonomic physiology and preceding events (Ekman and Cordaro, 2011). Indeed, discrete emotional expressions have yet to be developed during the first years of life. Moreover, the different emotional expressions seem to be advancing at different pace and to varying degrees impacted by social influences (Sullivan and Lewis, 2003). For instance, the “happy” expression develops from the newborn’s “sleepy smile” probably related to the discharge of pleasant stimulation by the infant’s still immature central nervous system, to a social smile by the age of 6 to 8 weeks. Social smiling peaks between 12 and 14 weeks, meanwhile other variants of “happy” expressions evolve, e.g., open-mouthed laughter or the non-social enjoyment of mastery (Sullivan and Lewis, 2003). While some cultural differences to “happy” expressions have been described, their general appearance seems to be universal (Camras et al., 1998). On the contrary, the development of the “surprise” expression seems to follow a different pattern. In very young infants, “surprise” is hardly observed at all, but at the latest by the age of 6 months at least a mild “surprise” expression appears as a reaction to novel events (Sullivan and Lewis, 2003). Yet, negatively toned “surprise” expressions or “surprise”/”fear” blends can be difficult to differentiate from “fear.”

Accordingly, in the studies employing images depicting infant facial expressions, whether for studying child development (Herba and Phillips, 2004; Strathearn et al., 2008), anxiety, depression, or trauma (Webb and Ayers, 2015) are often limited to a few emotions, often “happy” and “sad.” Further, these studies have relied on photos created specifically for the study, these images are not standardized, rated by a convenience sample (often students), and with respect to reproducibility most problematic: they are often not available to other researchers (Brosch et al., 2007; Thompson-Booth et al., 2014a,b). Thus, there is a great need of establishing standardized stimuli for measuring infant emotion processing.

Here, we introduce a standardized face database containing Caucasian infant face images from 18 infants. All infants display 4–7 facial expressions. Although Sullivan and Lewis (2003) argued that infants rarely show a “neutral” face before the age of 9 months, we did find “neutral” expressions and these are included in the database as they often serve as control stimuli. The photos were taken in a controlled environment by a professional photographer (LL). Validation data for the images is presented. For each image participants were asked to rate the depicted facial expression, and the clarity, intensity and valence of the expression. This allows researchers to select images with the specific properties required for their studies.

### Method

#### Development of the Image Set

**FIGURE 1:**
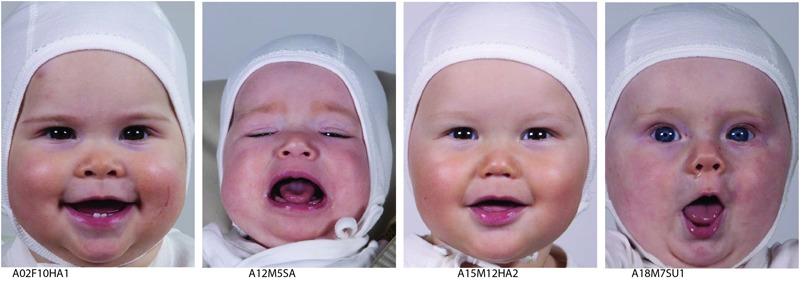
**Four of the 119 images from the database**.

The image set contains portrait images of 18 infants (10 female, 8 male) between the ages 4 and 12 months. There are 5–8 images of each baby displaying different emotional expressions (**Figure 1**). All infants wear a white infant bodysuit and a white hat in the images. This study was carried out in accordance with the recommendations of the Norwegian Data Protection Agency with written informed consent from all parents, in accordance with the Declaration of Helsinki. The protocol was approved by the Norwegian Data Protection agency, registration number 44418.

##### Recruitment

The families were recruited through maternity groups in social media, the university’s web page, and posters in health centers.

##### Procedure

Parents and their babies were welcomed by the photographer and introduced to the premises, the room where the photographing would take place and the nursery room. When all information was given and consent received from the parent, the parent changed the infant into the white infant bodysuit and hat provided. The infant was then placed in the infant chair and the photo session started. All photos were taken in the same room, against a uniform white background. Although infants show a variety of emotional expressions during 30 min of social interaction with a caregiver, we suggested some strategies to induce them. To induce happiness the parent was asked to play with and talk to the infant. An unfamiliar taste was used to induce the expression of disgust, for example a bit of lemon. Sadness occurred when the infant dropped a toy or got tired of the session. For ethical reasons considering the infants’ welfare we did not try to induce fear, surprise or anger during the session, although these expressions occurred naturally in some of the infants. The photographer took extra care in ensuring the infants’ wellbeing by suggesting breaks, encourage caring behavior from the parent, and being sensitive to the infants’ signals.

The session lasted 30–60 min. The parents received a gift card (500 NOK, approximately $60) after the session and some baby photos (without the white bodysuit and hat). In total, 19 sessions were completed, where two of them were test sessions, and one of them with twins. This resulted in images of 18 infants to be validated.

##### Pre-validation

The photographer first screened all photos for blurriness. In order to select the pictures for the validation study, all images where categorized by emotion by a male (DN) and two female researchers (AB, ÅL or LL, GP). The pictures with the highest inter-rater agreement were selected for the image set to be validated.

##### Image Processing

Pre-selected images were cropped and the eyes aligned and centered with a custom-written Matlab script. The final image size was 800 px by 1100 px. Finally, the photographer coded the images with a key that gave information about gender, age, time of photographing and what kind of consent was given. Name or other identifying information was not included in the file.

#### Validation of the Image Set

##### Participants

A total of 720 participants, distributed over five blocks ranging from 77 to 151 participants per block, were recruited for the validation study. A further 40 subjects did open the survey but never started it, i.e., 5% drop-out. Of all 720 participants, 53% did not complete the survey but over 80% of those rated at least 10 images and their data is included (see Supplementary Table S1 for different N per rated image). There were 79% female participants, and the mean age was 32.8 years (*SD* = 10.4, median = 31, range 18–70 years). Of all participants, 37% had children under the age of 3 years, 23% had children above the age of 3 years, 27% had no children and no regular contact with young children, and 13% had no children but regular contact with children under the age of 3 years. The participants were recruited online through social media, e-mail and the university’s website, where a short invitation and information about the study was published. Snowballing was an important method of recruiting.

Of each infant, six images were chosen for validation. Since we had not tried to induce surprise, fear and anger during the photo sessions, these expressions were less common. Based on the inter-rater agreement from the pre-validation we chose two images of happy expressions, one image of a sad expression, one image of a disgusted expression, one image of a neutral expression, and one depicting either surprise, fear or anger from each infant. In total, 153 images were presented for validation.

##### Procedure

The validation study was set-up online in Qualtrics^1^, allowing participants to answer the survey anonymously using their own computer or mobile device. In order to prevent drop-out by keeping the completion time at around 15–20 min, each participant was randomly assigned to validating a subset (block 1–3: 36 images; block 4: 25 images, block 5: 24 images) of the 153 images.

The first page described the purpose of the survey, contact information and instructions on the task. Participants were informed that they gave their consent by continuing to the next page, where demographic information was entered. On the next pages, participants were shown the infant pictures, one at the time, and asked to judge: (a) the depicted expression; (b) the clarity of the expression; (c) the intensity of the expression; and (d) the valence of the expression; in this order. In order to determine the predominant depicted expression, rating was forced-choice with eight response categories; the six basic emotions “happy,” “sad,” “surprised,” “disgusted,” “afraid” and “angry” and also “neutral” and “other.” We asked participants to pick the emotion label that best fitted the shown facial expression. When participants chose “other” they were asked to fill in what they would name the emotion in the picture. Participants were asked to rate the emotional expressions on 5-point Likert scales from “ambiguous” to “clear” (clarity), “weak” to “strong” (intensity), and from “very negative” to “very positive” (valence). In block 4 and 5 we removed the “other” option in the depicted expression question, as this had mostly served to collect synonyms of the six basic emotions in block 1–3. The questionnaire took about 18 min (block 1–3) or 13 min (block 4–5) to complete.

### Results

Only 24 participants (3%) indicated being in a sad mood, the majority, however, was in good or very good mood (79%).

In those five blocks, a total of 153 images were validated. We excluded 34 images with an average rating of clarity and intensity below 2.5. This left us with a database of 119 images.

#### Expression

We created an overview table of all images and their respective percentages of ratings (Supplementary Table S1) in the different emotion categories. The database contains the six basic emotions and neutral facial expressions, though not all infants have a complete set (see **Table 1**).

**Table 1:** Emotions depicted by each infant.

	Emotions						
Infant	Neutral	Happy	Sad	Disgusted	Angry	Afraid	Surprised
A02F10	x	xx	xo	o	xo	-	-
A03F07	xx	xo	x	x	-	-	xo
A04M06	xx	x	xx	x	-	-	x
A05F07	xx	xx	oo	o	SC	o	x
A06M05	xx	xx	x	-	x	-	-
A07M04	x	xx	x	x	-	x	-
A08M12	-	xx	oo	x	oo	o	x
A09F09	xx	xx	x	-	-	x	-
A10F05	x	xx	xx	-	x	-	x
A11F12	o	xx	x	x	x	-	xo
A12F05	xx	x	xoo	o	x	o	-
A13F05	xx	x	x	xo	o	-	-
A14M07	x	xo	xo	o	-	-	o
A15M12	x	xx	xx	-	-	-	x
A16F06	x	x	xx	o	o	xx	-
A17F09	x	xx	xo	x	-	o	xo
A18M07	x	xx	x	xo	-	o	xx
A19F06	xx	xx	x	-	x	-	x

*Number of images in database indicated by number of symbols (exception is “o” as many “o” can be one image); x, image rated as this particular emotion; xx, two images of this emotion included in the database; o, image rated as more than one emotion; SC, image rated by majority as “skeptical” according to “other” category; –, no image for this emotion. File name based on nomenclature by Lundqvist et al. (1998) and last two digits indicate age in months.*

#### Clarity, Intensity and Valence

The mean judgments for clarity, intensity and valence were calculated for each image (Supplementary Table S1). The overall means of the ratings on the three judgmental dimensions are displayed in **Table 2**.

**Table 2:** Average ratings (SDs) of clarity, intensity and valence per emotion.

	Emotion						
Measure	Happy	Sad	Disgust	Neutral	Fear	Anger	Surprise
	(*N* = 30)	(*N* = 20)	(*N* = 8)	(*N* = 25)	(*N* = 4)	(*N* = 6)	(*N* = 12)
Clarity	3.75 (0.5)	3.74 (0.4)	3.1 (0.3)	2.98 (0.2)	3.26 (0.2)	3.72 (0.3)	3.25 (0.3)
Intensity	3.55 (0.6)	3.89 (0.4)	3.36 (0.3)	2.80 (0.2)	3.53 (0.3)	3.97 (0.4)	3.33 (0.4)
Valence	4.05 (0.4)	2.01 (0.2)	2.39 (0.3)	3.02 (0.2)	2.13 (0.1)	2.11 (0.2)	3.02 (0.4)

*N referring to the number of images for each emotional expression.*

The valence scores show happiness as a clearly positive expression, whilst anger, sadness and fear were clearly negative. Disgust also came out as negative, although slightly less negative than the other negative emotions. Neutral came out as truly neutral, as well as surprise. All but the neutral expressions were rated as above average intense. During the photo session, all infants showed expressions where valence is rated as negative, positive and neutral.

### Discussion

The TIF database contains 119 images from 18 infants aged 4–12 months, thus being the first database providing high-quality, standardized images of facial expressions from this age group, for use in scientific research. The aim was to get four expressions from each infant: “happiness,” “sadness,” “disgust,” and “neutral.” As it turned out, several infants expressed anger, fear and surprise as well. Due to the nature of infants and their development, it was of course not possible to instruct them to hold still, focus their gaze and express the specific emotions needed. Neither did we wish to expose them to stimuli that would frighten them or cause them pain or discomfort. This might as well be a reason for the heretofore lacking of a validated infant facial expression database. However, an infant does show a range of emotional expressions within normal social interaction. This natural situation has resulted in a slightly different sample of expressions from each infant. From all infants we succeeded in getting at least four different expressions. According to the valence scores, all infants have expressed negative, positive and neutral emotions.

Previous studies have indicated that discrete, basic emotions yet have to develop during infancy and that infants generally express emotions less clear than older children and adults (Sullivan and Lewis, 2003; Camras and Shutter, 2010). Our findings suggest that infants by the age of 4–12 months show all the basic emotions described by Ekman and Cordaro (2011). Indeed, in term of recognition accuracy, emotions seem to be just as well recognizable in infants as they are in adults. Yet, as described by Sullivan and Lewis (2003), expressions are often blends of several emotions. In our study, discrete expressions were only for “happy.” Common blendings were “surprised”/”fear” and “sad”/”anger,” as found by Sullivan and Lewis (2003). Regarding “surprised,” previous studies indicate that the expression can rarely be seen until the age of 5–6 months (Sullivan and Lewis, 2003). Indeed, of the 18 infants in our sample, only seven showed “surprise.” Among the infants that where 6 month or less, only two displayed “surprise,” whereas of the infants that where 7–12 months old, only two did not show “surprise” during the photo session.

Further, Sullivan and Lewis (2003) claimed that infants rarely show “neutral” expressions before the age of 9 months. Yet, we found that all but one infant in our study showed expressions that were rated as “neutral.” These images were rated as perfectly “neutral” in terms of valence. They were rated as slightly less than “neutral” in terms of clarity and intensity, probably because they were perceived as more ambiguous and mild when compared to the pictures displaying the basic emotions. However, Sullivan and Lewis (2003) propose that infants most of the time show an “open interest” face, which could be directly related to the expression we propose is “neutral.”

In terms of validation, expression agreement varies across emotions and individual infants. Since the nature of photographing infants does not allow for instructions and practice, we could not ensure that the factorial combination of facial characteristics for each emotion was as standardized and clear as it is in the databases with adult faces. We therefore had to rely on the majority opinion of the validation to label what emotion was expressed in each image. For some images this resulted in a mix of two or three emotions. However, with an applied cut-off for clarity and intensity measure of larger than 3, respectively, we included these images. These less standardized expressions will also increase the ecological validity of the stimuli, as even adults rarely express emotions with a prototype facial expression in natural environments. The validation was done by a largely non-student sample, varying in their experience with childcare. To assess in more detail whether this experience influences emotion recognition in infants, we conducted a new study.

## Study 2: Infant Emotion Recognition by Experience

Primary caretaking of the infant is in many societies done by the mother or another female relative. This has led to the assumption that women might be better tuned than men to reading emotional expressions in infants (Babchuk et al., 1985; Cárdenas et al., 2013; Hahn et al., 2013). However, while behavioral studies sometimes find gender differences (Cárdenas et al., 2013; Hahn et al., 2013), others do not (Brosch et al., 2007; Parsons et al., 2011; Borgi et al., 2014). Neuroimaging evidence is similarly mixed with some studies indicating that women might be slightly more responsive to infant stimuli than men (Seifritz et al., 2003; Proverbio et al., 2006), whereas one study found the opposite (Weisman et al., 2012). Gender differences in emotion recognition in infant faces appear subtle and complex in nature. Instead of being mainly gender-specific, the ability to understand emotional expressions in infants may depend on experience. Babchuk et al. (1985) compared adults experienced in caretaking of infants to adults without such experience, with experience defined as contemporarily having children below the age of 5 years. They found that women even without experience with infants outperformed both fathers and non-fathers in terms of accuracy and speed in infant emotion recognition. However, the accuracy varied between emotions shown in the pictures. The difference was only significant for surprise, anger and marginally for fear, while the speed difference was only significant for surprise. Men were equally good as women in recognizing happy and sad, and both men and women had difficulties recognizing disgust. They concluded that these effects were independent of caregiving experience. More recently, Proverbio et al. (2007) investigated the effects of gender and expertise on the processing of valence and intensity of infant emotional expressions. Expertise was defined similarly as by Babchuk et al. (1985). Again, an advantage was found for women over men, especially for decoding the strong or weakly positive or weakly negative emotions. However, unlike Babchuk et al. (1985), they found that experience did affect accuracy in the decoding of emotions, but only in women. Mothers and women with regular contact with young children were more accurate in decoding strongly positive or negative emotions.

In contrast, one recent study by Parsons et al. (2016) did not find parents to be generally better than non-parents at recognizing infant emotions, yet found parents to be better tuned to facial expressions at low intensity, as in expressions close to neutral. Parents rated these neutral faces as less negative than non-parents. Interestingly, they found parenthood to affect men and women slightly differently. Where non-mothers and non-fathers did not differ in emotion recognition, significant differences were found between mothers and fathers. In particular, mothers rated positive expressions more positive than fathers and gave more extreme ratings for the most positive and most negative faces. These findings are compelling as they indicate that parenthood does have a subtle impact on emotion recognition of infant faces, yet suggest small gender differences regarding this influence. Still, several questions arise from these results. First, Parsons et al. (2016) included parents and non-parents in their study, but did not control for caretaking experience in the non-parent group. If the advanced emotion recognition abilities are due to experience rather than caused by biological and endocrine changes accompanying parenthood (Thompson-Booth et al., 2014b), adults without own children but with regular contact to infants should as well show differential emotion recognition. This could concern relatives, day-care personnel or other professionals working with children. Second, there is evidence suggesting that pregnant women already react as mothers when shown infant emotional expressions (Thompson-Booth et al., 2014b), whereas it is not known if becoming fathers perceive infant emotional stimuli already as fathers or still as non-fathers. Third, while Parsons et al. (2016) only examined the impact of parenthood on valence ratings of the infant stimuli, in the light of the outlined research it can also be expected that there might be differences in the intensity and clarity ratings of the emotion expressions.

Because the impact of gender and experience on infant emotion recognition remains elusive, it seemed desirable to examine the potential differences between male and female respondents with different relations to infants. In the current study, we investigated potential differences between men and women that either are parents of young or older children, those that are expecting their first baby, those that do not have any contact to small children at all and those that do not have own children but regularly are in contact with infants. In particular, we hypothesized that across all groups, accuracy, valence, intensity and clarity ratings depend on the respective emotion depicted. Regarding gender differences, we hypothesized that women give more extreme valence ratings and higher intensity ratings (Parsons et al., 2016), especially if they are mothers (Proverbio et al., 2007). Further, we hypothesized that clarity ratings depend on experience, and that parents judge “neutral” and “surprised” pictures as more clear than non-parents. Finally, across the dimensions, we hypothesized that first-time pregnant women rate the images more similarly to mothers than to non-mothers, whereas we expected the opposite pattern for first-time expecting fathers.

### Methods

#### Participants

A total of 1035 participants were recruited. Participants who completed less than 80% of the survey were excluded from data analysis, which left us with *N* = 421 (59% drop-out, counting every person that initially had opened the link to the survey). Of the remaining participants, 81% were women. The mean age was 30.25 years (*SD* = 7.3, median = 29, range 18–65). See **Table 3** for an overview over the groups and their demographic characteristics. In order to recruit a sufficient sample, the study was launched in both Norwegian and German^2^ language on social media, e-mail and the university’s website, where a short invitation letter with information about the study’s purpose and target group was provided^3^. In total, 60% of the participants reported that their nationality was German, while 31% of the participants were Norwegians. The remaining participants were from other German or Scandinavian speaking countries^4^. Again, to recruit parents of small children, pregnant women and expecting fathers, snowballing was an important method of recruiting. The study was carried out in accordance with the recommendations of the Norwegian Data Protection Agency with written informed consent from all subjects. All subjects gave informed consent in accordance with the Declaration of Helsinki^5^. The protocol was approved by the Norwegian Data Protection agency, registration number 49097.

**Table 3:** Participants by parental status and gender.

Group	No own children nor regular contact	Own children < 2 years	Own children > 2 years	First-time pregnant/expecting	Childless, but regular contact to children < 18 months
**Women**					
Number	72	99	80	28	62
Age	25.53 (4.14)	29.89 (4.50)	37.29 (8.34)	29.04 (6.13)	25.79 (5.74)
**Men**					
Number	25	22	17	11	5
Age	28.52 (6.38)	33.32 (7.64)	36.41 (5.57)	29.64 (4.39)	30.60 (6.99)

*Means for age in years are shown, with standard deviations in parentheses.*

#### Stimuli

The study contained 30 infant pictures that were predominantly displaying three of the six basic emotions (“happy,” “sad,” and “surprised”) in addition to “neutral.” The photographs were selected based on their ratings from Study 1, where the pictures with the highest participant agreement on the predominantly displayed emotion were chosen. For example, a “surprised” image could have been judged as “surprised” by 86% of the participants, and as “fear” or “happy” by the other 14% of participants. The participants’ agreement on the displayed emotion in each selected picture was 93 – 100% for “happy,” 69 – 79% for “sad,” 34 – 86% for “surprised” and 66 – 92% for “neutral.” In the selection process of “neutral” pictures, additional priority was given to capturing the whole range of valence as rated in Study 1. We restricted the study to these four emotions in order to get as many ratings as possible for the categories that are most often used in e.g., maternal depression studies (Strathearn et al., 2008; Laurent and Ablow, 2013; Thompson-Booth et al., 2014a).

#### Procedure

As in the validation study, we asked for taking part in a survey on understanding babies’ facial expressions. Participation was anonymous. The study was set up online in Qualtrics^6^ and assessed by the participants’ own computers or mobile devices.

Participants were asked to fill out demographic information, their current mood on a 5-point emoticon scale depicting a happy smiling, smiling, neutral, sad and very sad smiley. They were also asked if they were either pregnant or expecting father, or already had children. If they had children, they were asked if their youngest child was less than a year, less than 18 months, less than 2 years or more than 2 years old. To ensure that participants would not feel confused by the choices, there was also a choice for mothers and fathers who already had children and were expecting a new baby, in which case they also were asked for the age of their youngest child. If they had no children, they were asked if they were regularly in contact with children under the age of 18 months.

Participants were then shown the infant pictures, one at a time, and asked to judge: (a) the depicted expression; (b) the clarity of the expression; (c) the intensity of the expression; (d) the valence of the expression; (e) the genuineness of the expression; in this order. The depicted expression rating had seven response categories; “happy,” “sad,” “surprised,” “disgusted,” “fear,” “angry” and “neutral.” We asked participants to pick the emotion label that best fitted the shown facial expression. An “other” choice was not given as it in the validation study mainly had generated synonyms of the six basic emotions. The other four dimensions were rated as in Study 1, and genuineness from “not real” to “very real.” The questionnaire took on average 18 min to complete.

#### Data Analysis

We were not interested in nationality, and hence all reported analyses are pooled across the Norwegian and German version. We performed a 2 (gender female/male) by 5 (children < 2 years, children > 2 years, first child expecting, no children, no children but regular contact with children < 1.5 years) by 4 (happy, neutral, sad, surprise) mixed analysis of variance. Mood was a covariate in all analyses. We separately looked at accuracy, valence, intensity and clarity. We also measured the time required to answer each item, that is, how long the respondents needed to answer the five questions after seeing the image. Response times over 6 min were excluded as these might be due to disruptions (online survey). Genuineness was calculated over all groups per image, as we were only interested in the average score. We report Greenhouse–Geisser corrected values, since Mauchly’s test of sphericity was significant. When looking at one emotion only, we corrected for multiple comparison, i.e., we applied a conservative significance criterion of *p* < 0.0125.

### Results

Demographic characteristics are summarized in **Table 3**. Mean ratings were obtained for all groups and each emotion on all dimensions (valence, intensity, clarity and genuineness).

The average rating for mood was 4.00 (*SE* = 0.55). None of the participants reported a mood lower than 2. The average response times for happy images was 32 s, for neutral 40 s, for sad 32 s and for surprise 41 s.

All images received high genuineness ratings; averages ranged from 3.85 to 4.57. Supplementary Table S2 provides the genuineness rating for each image.

#### Accuracy

**FIGURE 2:**
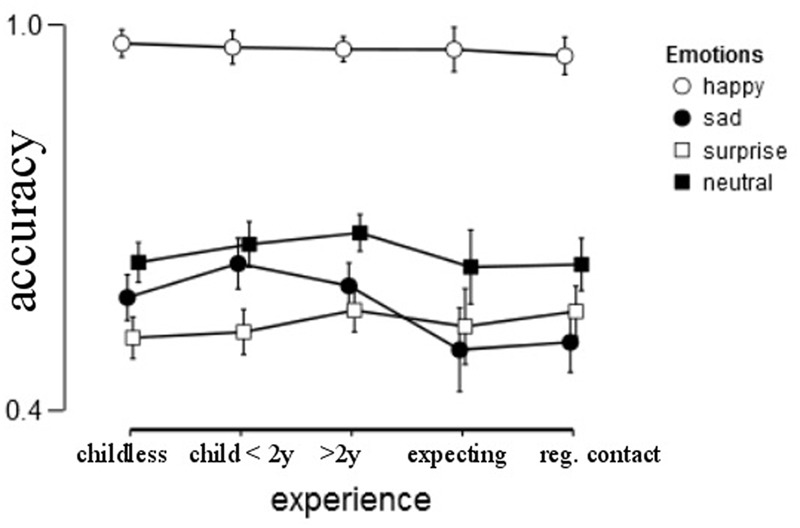
**Average accuracy by group for each of the four emotions.** Error bars represent 95% confidence interval.

Emotion recognition accuracy depended profoundly on the emotion shown [*F*(2.546,1043.887) = 22.788, *p* < 0.001, $\eta_{p}^{2}$ = 0.053). “Happy” was easily recognized (94 – 100%), whereas “sad” (44 – 65%), “surprise” (48 – 63%) and “neutral” (60 – 77%) were somewhat harder to judge from static pictures (**Figure 2**).

We predicted that accuracy in emotion recognition does not depend on gender, but that the recognition depends on experience. Indeed, no gender differences were found [*F*(1,410) = 0.433, *p* = 0.511, $\eta_{p}^{2}$ = 0.001]. The expected effect of experience was not found either [*F*(4,410) = 2.313, *p* = 0.057, $\eta_{p}^{2}$ = 0.022]. Mood did not influence accuracy, *F*(1,410) = 2.553, *p* = 0.111, $\eta_{p}^{2}$ = 0.006. There was no interaction between gender and experience [*F*(4,410) = 1.506, *p* = 0.200, $\eta_{p}^{2}$ = 0.014], nor between the emotion factor and gender (*p* > 0.8), emotion factor and experience (*p* = 0.066) and the three-way interaction emotion factor by gender by experience (*p* > 0.36).

Since previous studies found a difference for sad stimuli by experience, we looked separately at the accuracy of sadness. This ANCOVA yielded a main effect for experience, *F*(4,410) = 3.707, *p* = 0.006, $\eta_{p}^{2}$ = 0.035. *Post hoc* Tukey yielded a group difference between those with children and pregnant/first expecting parents, (*p* = 0.016 for comparison group children < 2 years versus pregnant and *p* = 0.033 for comparison children > 2 years versus pregnant). There was no main effect of gender, *p* = 0.925, nor an interaction between experience and gender, *p* = 0.732. The covariate mood was not significant either, *p* = 0.062. A similar analysis for the other three emotions did not yield a main effect of experience on accuracy. An analysis by re-grouping the groups in parents and non-parents yielded also no statistically significant effects.

#### Valence

As expected, valence ratings depended on the emotion shown [*F*(1.829,201.797) = 67.354, *p* < 0.001, $\eta_{p}^{2}$ = 0.139]. “Sad” was rated as negative (*M* = 1.93, *SD* = 0.70), “surprise” and “neutral” were similarly rated as slightly more negative than positive (*M* = 2.90, *SD* = 0.47 and *M* = 2.96, *SD* = 0.38, respectively), and “happy” was rated as positive (*M* = 4.54, *SD* = 0.46).

**FIGURE 3:**
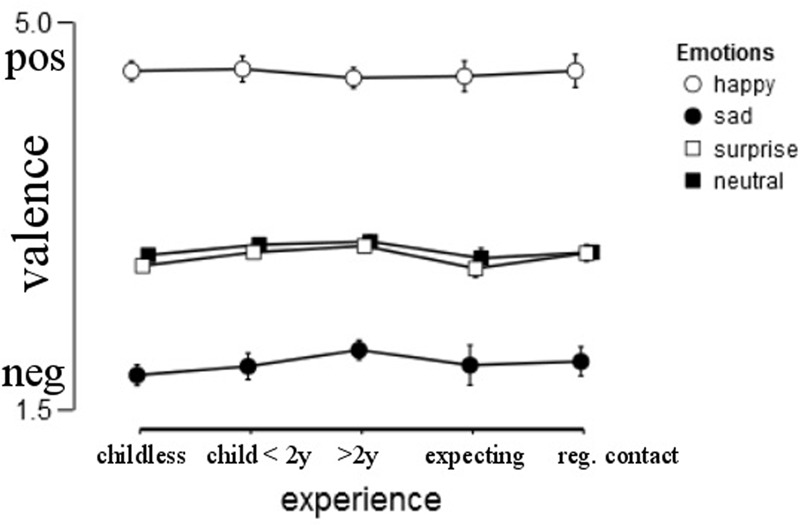
**Average valence rating by group, error bars represent 95% confidence interval**.

Overall, there was no significant effect of gender on the valence ratings [*F*(1,403) = 1.007, *p =* 0.316, $\eta_{p}^{2}$ = 0.002]. Experience did not influence valence ratings, *F*(4,403) = .974, *p* = 0.421, $\eta_{p}^{2}$ = 0.009, nor did mood, *F*(1,403) = 1.274, *p* = 0.260, $\eta_{p}^{2}$ = 0.003. The interaction between gender and experience was also not significant, *F*(4,403) = 1.476, *p* = 0.209, $\eta_{p}^{2}$ = 0.014, nor was any of the other interactions, all *p*s > 0.05 (**Figure 3**).

To test the hypothesis that women give more extreme valence ratings for positive and negative emotions, we specifically looked for gender effects on the “happy” and “sad” stimuli. While women indeed gave significantly more extreme ratings (women *M* = 4.5, *SD* = 1.41; men *M* = 4.39, *SD* = 0.61) for positive (“happy”) pictures [*F*(1,408) = 6.945, *p* = 0.009, $\eta_{p}^{2}$ = 0.017], this was not the case for negative (“sad”) (women: *M* = 1.92, *SD* = 0.74, men: *M* = 1.96, *SD* = 0.56) pictures [*F*(1,404) = 0.392, *p* = 0.531, $\eta_{p}^{2}$ = 0.001].

Additionally, we predicted that parents (*M* = 2.99, *SD* = 0.41) would rate “neutral” expressions more positive than non-parents (*M* = 2.89, *SD* = 0.32), which was supported [*F*(1,411) = 5.785, *p* = 0.017, $\eta_{p}^{2}$ = 0.014].

#### Intensity

**FIGURE 4:**
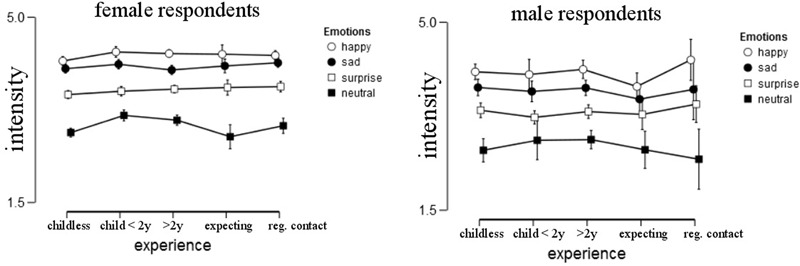
**Intensity ratings per gender and group, error bars represent 95% confidence intervals**.

Intensity ratings depended on the emotion shown [*F*(2.668,195.767) = 27.821, *p* < 0.001, $\eta_{p}^{2}$ = 0.063]. Of all emotions, “happy” was rated the most intense (*M* = 4.24, *SD* = 0.55), followed by “sad” (*M* = 4.0, *SD* = 0.55), “surprise” (*M* = 3.56, *SD* = 0.59) and “neutral” (*M* = 2.92, *SD* = 0.71). *Post hoc* comparisons using the Tukey HSD test indicated that the mean scores were significantly different for all emotions (*ps* < 0.001) (**Figure 4**).

We hypothesized that women give higher intensity ratings than men. Indeed, we found a significant main effect of gender, with women (*M* = 3.74, *SD* = 0.59) rating the images as more intense than men [*M* = 3.46, *SD* = 0.61; *F*(1,403), *p* < 0.001, $\eta_{p}^{2}$ = 0.038].

There was neither a main effect of experience [*F*(4,403) = 0.401, *p* = 0.808, $\eta_{p}^{2}$ = 0.004] nor an interaction between gender and experience on intensity ratings [*F*(4,403) = 0.421, *p* = 0.793, $\eta_{p}^{2}$ = 0.004], but there was an influence of mood on intensity ratings, *F*(1,403) = 9.403, *p* = 0.002, $\eta_{p}^{2}$ = 0.022. The happier the participant the more intense the emotion rating was.

When specifically comparing parents and non-parents we found a significantly higher intensity rating for parents [*F*(1,411) = 4.832, *p* = 0.028, $\eta_{p}^{2}$ = 0.012], and a significant interaction between emotions and experience, *F*(2.666,200.401) = 3.857, *p* = 0.012, $\eta_{p}^{2}$ = 0.009. This effect was driven by a more intense rating of neutral stimuli in parents than non-parents.

#### Clarity

Judging the clarity of emotional expressions depends on the respective emotion [*F*(2.859,260.965) = 33.324, *p* < 0.001, $\eta_{p}^{2}$ = 0.075]. “Happy” expressions were rated the clearest (*M* = 4.42, *SD* = 0.55), followed by “sad” and “surprised” expressions (*M* = 3.64, *SD* = 0.75 and *M* = 3.33, *SD* = 0.72, respectively) and “neutral” expressions were rated the least clear (*M* = 3.07, *SD* = 0.76).

We found a significant main effect of gender, with women giving higher clarity ratings than men [*F*(1,403) = 5.167, *p* = 0.024, $\eta_{p}^{2}$ = 0.012].

Experience did not influence clarity ratings [*F*(4,403) = 1.007, *p* = 0.404, $\eta_{p}^{2}$ = 0.010]. Mood had a significant effect on clarity, *F*(1,403) = 7.337, *p* = 0.015, $\eta_{p}^{2}$ = 0.014. None of the interactions were significant, all *ps* > 0.05. With respect to “neutral” images, we found that parents (*M* = 3.16, *SD* = 0.8) rated neutral images as clearer than non-parents (*M* = 2.89, *SD* = 0.65), *F*(1,411) = 8.988, *p* = 0.003, $\eta_{p}^{2}$ = 0.021. Parenthood was a stronger predictor than mood, *F*(1,411) = 7.523, *p* = 0.006, $\eta_{p}^{2}$ = 0.018. Similarly, sad images were rated as more clearly in parents (*M* = 3.72, *SD* = 0.77) than in non-parents (*M* = 3.49, *SD* = 0.68), *F*(1,412) = 6.861, *p* = 0.009, $\eta_{p}^{2}$ = 0.016. The covariate mood did not reach the more stringent significance criterion, *F*(1,412) = 5.367, *p* = 0.021, $\eta_{p}^{2}$ = 0.013.

**FIGURE 5:**
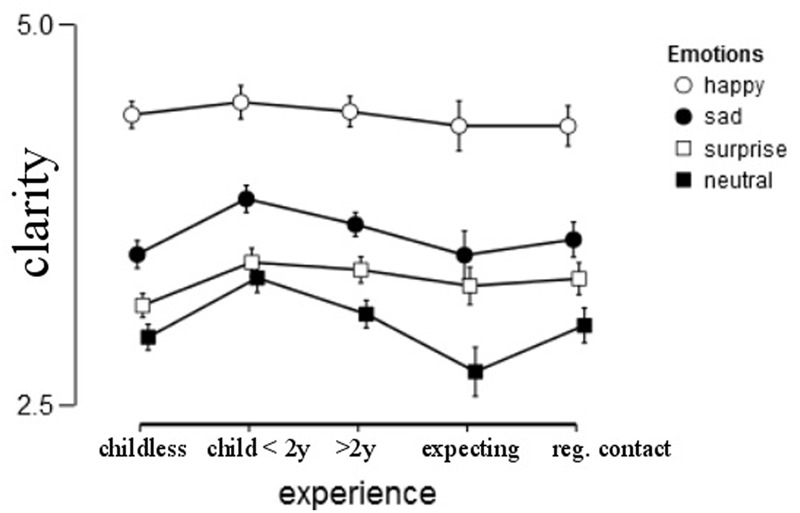
**Average clarity ratings per group, error bars represent 95% confidence intervals**.

The main effect of experience did not reach significance for “surprise,” i.e., *F*(1,414) = 5.8, *p* = 0.016, $\eta_{p}^{2}$ = 0.014 but also here parents (*M* = 3.4, *SD* = 0.76) rated the images as more clearly than non-parents (*M* = 3.19, *SD* = 0.62). There was no difference for happy images, all *ps* > 0.35 (**Figure 5**).

There was a strong positive correlation between intensity and clarity ratings, *r* = 0.77. Further, for valence, intensity and clarity the intraclass correlation (ICC) using a consistency definition was 0.884 (including accuracy reduced the ICC to 0.864).

### Discussion

The aim of this study was to examine the impact of gender and experience on the recognition of emotions in infant faces. As infants communicate most of their physical and psychological needs through crying and facial expressions, it is of major importance to assess whether there are differences in the ability to read their facial expressions and hence their emotional state. We found that emotion recognition accuracy as well as valence, intensity and clarity ratings depend on the emotion shown. Overall, accuracy in emotion recognition in the infant faces was similar to accuracy in the recognition of expressions in adult faces (Keltner and Cordaro, 2015). Neither gender nor experience and mood influenced accuracy in judgments. Experience may weakly influence accuracy judgments, i.e., first time expecting parents were less often judging “sad” stimuli as “sad” compared to parents. However, this difference did not remain when we grouped expecting parents with those that have no children. Similarly, overall valence ratings were not influenced by gender, although women rated “happy” faces as slightly more positive than men. Further, experience or mood had no impact on valence ratings. Intensity ratings, though, were strongly influenced by gender and mood, but not by experience. As found in a previous study (Parsons et al., 2016), women gave more extreme ratings than men. Finally, clarity ratings were influenced by both gender and mood. Comparing parents to non-parents we found slightly more intense ratings in parents for sad and neutral facial expressions (**Table 4**). There was a strong positive correlation between intensity and clarity ratings, indicating consistency across both scales. Finally, the ICC for valence, intensity and clarity indicates consistency within participants.

**Table 4:** Overview over the results, displayed by gender/parenthood and emotion.

	Happy	Sad	Surprise	Neutral
Accuracy	♀ = ♂	♀ = ♂	♀ = ♂	♀ = ♂
Valence	♀ > ♂	♀ = ♂	♀ = ♂	♀ = ♂
Intensity	♀ > ♂	♀ > ♂	♀ > ♂	♀ > ♂, P > Non-P
Clarity	♀ > ♂	♀ > ♂, P > Non-P	♀ > ♂	♀ > ♂, P > Non-P

*P, parent; Non-P, non-parent.*

Our first hypothesis was that accuracy, valence, intensity and clarity ratings depend on the emotion shown. The results support that, as did results from previous studies (Babchuk et al., 1985). This finding is reassuring, because it supports our premise that the stimuli used indeed depicture distinct emotions with their individual properties. Still, unlike adult faces, infant faces are characterized by the baby schema (Lorenz, 1943, 1971) and infants can express emotions less clearly than adults (Camras and Shutter, 2010). Because mothers or another female relative traditionally have had the primary caregiving responsibility, it has been assumed that women might be better tuned to reading emotional expressions in infants (Babchuk et al., 1985; Cárdenas et al., 2013; Hahn et al., 2013). Yet, as the basic emotions are widely accepted to be universal in nature (Darwin, 1872/1998; Ekman, 2016; Ekman and Friesen, 1969), we did neither expect nor find a gender effect in terms of decoding accuracy. Two earlier studies did find a small advantage for women in emotion recognition accuracy (Babchuk et al., 1985; Proverbio et al., 2007), but these findings were based on smaller samples, less validated images and not consistent when compared to each other. However, while it is a reassuring finding that men and women seem to be equally well equipped to decode basic emotional expressions in infant faces, women might be better tuned to perceiving nuances in the expression. Proverbio et al. (2007) proposed that women extract affective information from the infant face guided by subtle differences in arousal.

Thus, our second hypothesis was that women give more extreme valence ratings and higher intensity ratings, especially if they are mothers. We did indeed find small gender differences in terms of valence, intensity and also clarity ratings. For valence ratings, there was no overall effect of gender, but women rated positive images as more positive than men did. Moreover, regardless of their children’s age, mothers rated the images as more positive than non-mothers, whereas there was no such difference between fathers and non-fathers. This is consistent with findings reported by Parsons et al. (2016), who subsequently suggest that parenthood affects men and women differently. Additionally, women gave higher ratings of both intensity and clarity. The gender effect was the most pronounced for intensity ratings. Women giving more extreme intensity ratings is consistent with the finding by Proverbio et al. (2007) that women were better than men at decoding the strongest infant emotions.

Our third hypothesis was that clarity ratings depended on experience, which was only weakly supported by our data. Those that currently have or previously had experience in caretaking of infants rated “neutral” and “sad” images as slightly more clearly than did non-parents, including first-time expecting parents. Despite the higher clarity ratings related to caretaking experience, accuracy in emotion recognition was not generally increased for experienced caretakers, although parents of young children were better at detecting “sad” expressions. Likewise, two previous studies found no effect of experience on emotion recognition accuracy (Babchuk et al., 1985) or an experience effect on women’s emotion recognition ability only (Proverbio et al., 2007). One possible explanation for the increase in clarity, but not accuracy, for parents of young children might be that they implicitly understand the expression and know how to react to it, but without explicitly being able to name it. For example, Beebe and Lachmann (2002) found that caretakers and newborns within a fraction of a second attuned to each other’s emotional expressions, which is much faster than a conscious, explicit understanding of the expression could be established. In the current study, this implicit “clarity” of infant emotions perceived by parents of young children stands out as the strongest impact of caretaking experience. Other studies have shown that caregiving experience seems to influence valence ratings (Parsons et al., 2016), the ability to recognize the own infant from their cry (Gustafsson et al., 2013) as well as neural activity in the amygdala and interconnected limbic activation in response to infant crying (Seifritz et al., 2003) and the strength of the “caregiving neural circuit” (Abraham et al., 2014).

Further, our fourth hypothesis was that parents are better able to identify “neutral” and “surprised” pictures than non-parents. Indeed, parents rated “surprised” images as clearer, and “neutral” images as slightly more positive, more intense and clearer than non-parents. Regarding valence ratings, Parsons et al. (2016) found the same pattern. As parents, consistently with our findings rated the images closer to neutral than non-parents, they suggested that parents might indeed be better attuned to ambiguous emotional expressions such as “neutral,” when compared to non-parents. This is also in line with our finding that caretaking experience was found to be associated with higher clarity ratings in general. However, this finding is restricted to parenthood and did not generalize to participants that are in regular contact with infants.

Finally, our last hypothesis was that across all dimensions, first-time pregnant women rate the images more similarly to mothers than to non-mothers, whereas we expected first-time expecting fathers to rate the images more similarly to non-fathers. We did not find the expected pattern, as first-time pregnant women on most dimensions rated the images similarly as non-mothers. This could be due to the relatively low number of pregnant women in the study. Additionally, we did not control for the pregnancy month, where processing differences due to neuroendocrine changes could be expected to be more salient in late pregnancy (Russell et al., 2001).

An important covariate was mood. We included a quick self-judged mood rating since it has been shown that emotion recognition is influenced by one’s mood (Parsons et al., 2016). Intensity and clarity were affected by a participants’ mood, whereas accuracy and valence were not affected. Thus, we recommend controlling for mood when investigating facial emotional processing.

### Limitations

The majority of participants were women. Also, we did not include age as a covariate. Recently, Hartshorne and Germine (2015) have reviewed that face recognition is improving until the age of 50, based on studies using mainly adult faces. In this study ‘experience’ was from a theoretical point of view more important than participant’s age. Yet, in the questionnaire the quantity of ‘experience’ was not sufficiently specified and the small ‘experience’ effect could be biased by some participants with relatively little caretaking experience responding ‘yes’ on the relevant item. Also, to disentangle age from experience a more controlled recruiting of young and late parents would be required. Further, as the TIF database only contains Caucasian infants, the findings could be less applicable for non-Caucasian target groups (Russell, 1994). Moreover, the participants were presented with unfamiliar infant photographs only. While this yields important information about the ability to read unfamiliar infant faces, it has previously been found that emotion recognition in the own infant is even more fine-tuned (Strathearn et al., 2008). Further, all rating occurred on static images without any context provided. This rather artificial situation may engage different brain processes than a social, 3D sensory rich interaction. However, due to their applicability, photographs are more often used for research purposes than dynamic photo material. Finally, in order to induce a “disgust” expression in the infants, they were given lemon juice to taste. Yet, Sullivan and Lewis (2003) note that the response to sour taste is more variable and milder when compared to the response to a bitter taste, which thus could have been a more suitable stimulus.

### Implications

The finding that experience seems to enhance clarity ratings of infant emotional expressions is important, as this could be related to an implicit understanding of how to appropriately react to the infant. How perceived clarity affects caregiving interactions would hence be an important question for further research. Moreover, clarity could even be a more relevant and valid dimension in emotion recognition in infants than valence and intensity ratings. This notion is supported by the fact that several respondents contacted us during data collection and expressed confusion about the difference between valence and intensity.

## Conclusion

In Study 1, we presented the development and validation of the TIF database. The TIF database contains 119 high-quality images from infants aged 4–12 months old, thus being the first standardized database for images of emotional expressions from this age group. Further, the study yielded all the six basic emotions in young infants, extending previous studies that used one category of negative valence: distress. In Study 2, we used a selection of 30 images from the TIF database to investigate differences in the emotion recognition accuracy, as well as in the clarity, intensity and valence ratings when comparing men and women at different stages of parenting. Additionally, Study 2 provided a stable replication of Study 1, as those 30 images on average have received similar ratings as found in Study 1 and were rated as highly genuine by all participants.

Both studies show that although infants due to fat tissue and facial muscles express their emotions less clearly than adults (Camras and Shutter, 2010), adults independently of caretaking experience and gender generally seem to understand infant facial expressions just as well as adult facial expressions (Keltner and Cordaro, 2015). While we did find a small gender effect in terms of women providing higher intensity and clarity ratings as well as more positive ratings of happy images, even more interesting is that experience seems to enhance clarity ratings for neutral, sad, and surprise images. We recommend to use “neutral” images to achieve higher discriminability among raters.

## Database Access

The database can be found at http://site.uit.no/sin/tromso-infant-faces-database/ and entering the password UiT_TIF2015.

## Author Contributions

GP designed the study, LL recruited families with infants and photographed them, MØ created the images, AB, ÅAWL, CEAW, DN, GP, and LL prescreened the images. JKM and AB created the surveys. All authors recruited respondents for the surveys. JKM, AB, and GP analyzed the data. JKM, AB, GP, DN, and CEAW wrote the manuscript.

## Conflict of Interest Statement

The authors declare that the research was conducted in the absence of any commercial or financial relationships that could be construed as a potential conflict of interest.

## Footnotes

1 Qualtrics.com

2 Two of the authors are native German speakers as well as fluent in Norwegian.

3 Over 90% of respondents were from Norway and Germany, and in both countries fathers can take a paternal leave.

4 Three from Sweden, two from Denmark, 13 from Austria, six from Switzerland, one from Lichtenstein, 13 indicated currently residing in another country than those 7 seven specified.

5 The online survey stated that starting the survey meant consent given.

6 Qualtrics.com
