# Supplementary material for: The Tromso Infant Faces Database (TIF): Development, Validation and Application to Assess Parenting Experience on Clarity and Intensity Ratings
Source: Front Psychol. 2017 Mar 24;8:409. doi: 10.3389/fpsyg.2017.00409 (PMC5364182; doi:10.3389/fpsyg.2017.00409)
Supplement: Supplementary file 1 [file Table_1.docx]

Supplementary table 1: Validation data for the 119 images included in the TIF database

| **Infant** | **Image** | **Happy** | **Sad** | **Disgust** | **Anger** | **Fear** | **Surprise** | **Neutral** | **Other** | **Clarity (M & SD)** | | **Intensity (M & SD)** | | **Valence (M & SD)** | | **Respondents** |
| --- | --- | --- | --- | --- | --- | --- | --- | --- | --- | --- | --- | --- | --- | --- | --- | --- |
| **A02** | A02F10HA1 | 91% | - | - | - | - | 8% | 1% | - | 4.20 | 0.92 | 3.91 | 0.91 | 4.39 | 0.73 | 98 |
|  | A02F10HA2 | 96% | - | - | - | - | 4% | - | - | 4.75 | 0.68 | 4.70 | 0.61 | 4.84 | 0.55 | 113 |
|  | A02F10SA | - | 57% | 4% | 14% | 21% | 1% | - | 2% | 3.66 | 0.98 | 3.76 | 0.81 | 2.13 | 0.82 | 93 |
|  | A02F10AN | - | 11% | 13% | 66% | 9% | - | 1% | 1% | 3.77 | 1.02 | 3.98 | 0.80 | 2.05 | 1.05 | 119 |
|  | A02F10NE | - | 4% | 7% | 18% | 1% | - | 70% |  | 3.04 | 1.13 | 2.84 | 1.08 | 2.88 | 0.73 | 77 |
|  | A02F10DI-SA-AN | - | 27% | 36% | 24% | 11% | 2% | - |  | 3.24 | 1.15 | 3.65 | 0.88 | 2.48 | 1.06 | 63 |
| **A03** | A03F7HA-SU | 55% | - | - | - | - | 43% | - | 2% | 3.91 | 0.92 | 3.82 | 0.82 | 4.28 | 0.76 | 92 |
|  | A03F7SA | - | 71% | 1% | 15% | 12% | - | - | 1% | 3.91 | 1.07 | 4.05 | 0.82 | 2.07 | 1.03 | 136 |
|  | A03F7SU | - | - | - | - | 1% | 64% | 32% | 3% | 3.26 | 1.08 | 3.09 | 1.06 | 3.33 | 0.75 | 96 |
|  | A03F7HA | 57% | - | - | 1% | - | 39% | - | 3% | 3.87 | 0.89 | 3.63 | 0.83 | 4.32 | 0.63 | 115 |
|  | A03F7NE1 | 3% | - | - | - | - | 24% | 73% |  | 3.41 | 1.12 | 3.16 | 1.15 | 3.32 | 0.64 | 63 |
|  | A03F7NE2 | - | 11% | 23% | 2% | 4% | 5% | 56% |  | 2.87 | 1.17 | 2.81 | 1.01 | 2.96 | 0.67 | 54 |
|  | A03F7DI | - | 18% | 58% | 1% | 8% | 14% | 1% |  | 2.93 | 1.23 | 3.40 | 0.96 | 2.32 | 0.79 | 75 |
| **A04** | A04M6SA1 | - | 66% | 6% | 6% | 22% | - | - | 1% | 3.90 | 1.04 | 4.05 | 0.81 | 2.13 | 1.08 | 100 |
|  | A04M6DI | - | 19% | 46% | 4% | 11% | 3% | 12% | 5% | 2.84 | 1.15 | 3.06 | 1.00 | 2.46 | 0.81 | 110 |
|  | A04M6NE1 | - | 20% | 2% | 2% | 4% | 2% | 54% | 17% | 2.79 | 1.06 | 2.63 | 0.98 | 2.88 | 0.70 | 117 |
|  | A04M6NE2 | 6% | 4% | 1% | - | 3% | 3% | 71% | 13% | 2.86 | 1.08 | 2.50 | 1.02 | 3.07 | 0.70 | 129 |
|  | A04M6HA | 99% | - | - | - | - | - | - | 1% | 4.71 | 0.60 | 4.60 | 0.65 | 4.77 | 0.48 | 91 |
|  | A04M6SU | - | 4% | 3% | - | 17% | 74% | 3% |  | 3.55 | 1.08 | 3.63 | 0.81 | 2.97 | 0.84 | 71 |
|  | A04M6SA2 | 1% | 59% | 8% | 3% | 29% | - | - |  | 3.12 | 1.09 | 3.37 | 0.97 | 2.19 | 0.93 | 75 |
| **A05** | A05F7HA1 | 83% | - | - | - | 1% | 4% | 9% | 4% | 3.67 | 1.01 | 3.56 | 0.91 | 4.00 | 0.77 | 81 |
|  | A05F7DI-SA | - | 31% | 46% | 16% | 2% | 1% | 0% | 4% | 3.78 | 1.11 | 3.86 | 0.96 | 2.18 | 0.95 | 80 |
|  | A05F7AF-SA | - | 43% | 1% | 1% | 51% | 1% | - | 2% | 3.81 | 1.17 | 4.13 | 0.92 | 2.07 | 1.15 | 85 |
|  | A05F7SU | - | 3% | 4% | 7% | 20% | 34% | 21% | 11% | 2.93 | 1.14 | 3.00 | 1.06 | 2.84 | 0.67 | 110 |
|  | A05F7HA2 | 88% | - | - | 1% | 2% | 4% | 2% | 3% | 4.02 | 0.99 | 3.92 | 0.92 | 4.06 | 0.94 | 95 |
|  | A05F7SC | - | 2% | 18% | - | 26% | 12% | 6% | 37% * | 3.03 | 1.05 | 3.06 | 0.97 | 2.62 | 0.73 | 117 |
|  | A05F7NE1 | - | 11% | 11% | 11% | 13% | 11% | 45% |  | 2.81 | 1.13 | 3.15 | 1.08 | 2.70 | 0.77 | 53 |
|  | A05F7NE2 | - | 4% | 7% | - | 11% | 18% | 60% |  | 2.92 | 1.15 | 2.92 | 1.04 | 2.83 | 0.76 | 52 |
| **A06** | A06M5HA1 | 67% | 3% | - | - | 1% | 2% | 17% | 11% | 3.37 | 1.06 | 2.84 | 1.08 | 3.86 | 0.79 | 128 |
|  | A06M5SA | - | 63% | 3% | 1% | 23% | - | 1% | 9% | 3.51 | 1.03 | 3.43 | 0.85 | 2.11 | 0.77 | 115 |
|  | A06M5NE1 | - | 1% | - | - | 2% | 5% | 91% | - | 3.28 | 1.09 | 2.79 | 1.08 | 3.09 | 0.48 | 80 |
|  | A06M5AN | - | 17% | 5% | 52% | 18% | - | 1% | 8% | 3.59 | 1.07 | 3.73 | 0.89 | 2.23 | 0.97 | 105 |
|  | A06M5HA2 | 100% | - | - | - | - | - | - | - | 4.35 | 0.79 | 3.92 | 0.93 | 4.35 | 0.74 | 88 |
|  | A06M5NE2 | 22% | - | 15% | - | - | - | 64% |  | 3.19 | 1.16 | 2.75 | 1.08 | 3.35 | 0.74 | 52 |
| **A07** | A07M4HA1 | 75% | - | 1% | - | - | 12% | 5% | 7% | 3.26 | 1.16 | 3.00 | 0.99 | 3.75 | 0.87 | 97 |
|  | A07M4AF | - | 15% | 14% | 2% | 50% | 6% | 3% | 11% | 3.08 | 1.06 | 3.16 | 1.01 | 2.09 | 0.67 | 99 |
|  | A07M4NE | 1% | 5% | 3% | 2% | 3% | 12% | 70% | 5% | 2.86 | 1.15 | 2.64 | 1.02 | 2.99 | 0.59 | 101 |
|  | A07M4DI | 1% | 10% | 47% | 20% | 5% | 3% | 4% | 10% | 3.67 | 1.02 | 3.95 | 0.94 | 1.96 | 0.85 | 107 |
|  | A07M4HA2 | 49% | 7% | 2% | 1% | - | 25% | 9% | 7% | 2.89 | 1.12 | 2.87 | 0.98 | 3.45 | 0.78 | 135 |
|  | A07M4SA | - | 74% | 14% | 9% | 4% | - | - |  | 3.72 | 1.05 | 4.09 | 0.85 | 1.87 | 0.93 | 54 |
| **A08** | A08M12HA1 | 77% | - | 1% | - | 1% | 10% | 6% | 6% | 3.26 | 1.14 | 2.96 | 0.98 | 3.85 | 0.74 | 135 |
|  | A08M12DI | - | 22% | 40% | - | 1% | 7% | 23% | 6% | 2.73 | 1.19 | 2.83 | 1.07 | 2.59 | 0.83 | 109 |
|  | A08M12SU | - | 11% | 13% | - | 25% | 47% | 1% | 3% | 3.15 | 1.00 | 3.45 | 0.83 | 2.45 | 0.72 | 92 |
|  | A08M12HA2 | 73% | - | - | - | 2% | 24% | 1% | - | 3.59 | 1.22 | 3.55 | 1.02 | 4.03 | 0.75 | 92 |
|  | A08M12AN-SA | - | 37% | 2% | 45% | 13% | - | - | 3% | 4.55 | 0.76 | 4.83 | 0.40 | 1.55 | 1.20 | 109 |
|  | A08M12AF-SA-AN | - | 26% | 4% | 25% | 44% | 2% | - |  | 3.89 | 1.04 | 4.33 | 0.87 | 1.47 | 0.67 | 54 |
| **A09** | A09F9HA1 | 77% | 2% | - | - | - | 3% | 14% | 5% | 3.33 | 1.04 | 2.93 | 0.94 | 3.78 | 0.72 | 150 |
|  | A09F9SA | - | 79% | 1% | 4% | 15% | - | - | 2% | 4.45 | 0.78 | 4.54 | 0.63 | 1.81 | 1.09 | 128 |
|  | A09F9NE1 | - | 17% | 1% | 1% | 3% | 1% | 75% | 3% | 3.25 | 1.11 | 2.82 | 0.99 | 2.78 | 0.61 | 110 |
|  | A09F9NE2 | - | 15% | 4% | - | 3% | 13% | 59% | 7% | 2.90 | 1.08 | 2.84 | 0.91 | 2.94 | 0.55 | 100 |
|  | A09F9HA2 | 52% | 6% | 1% | - | 2% | 4% | 31% | 4% | 2.90 | 1.05 | 2.67 | 0.95 | 3.39 | 0.70 | 97 |
|  | A09F9AF | 3% | 11% | 2% | 2% | 73% | 10% | - |  | 3.20 | 1.30 | 3.53 | 1.02 | 2.23 | 0.96 | 60 |
| **A10** | A10F5HA1 | 78% | - | - | - | - | 1% | 14% | 7% | 3.60 | 1.03 | 2.95 | 1.17 | 3.95 | 0.76 | 84 |
|  | A10F5AN | - | 17% | 14% | 61% | 5% | 1% | - | 2% | 4.05 | 1.01 | 4.45 | 0.70 | 2.14 | 1.30 | 85 |
|  | A10F5NE | 3% | 1% | - | - | 1% | 1% | 83% | 11% | 3.52 | 1.09 | 2.93 | 1.10 | 3.29 | 0.65 | 134 |
|  | A10F5SA1 | - | 77% | 1% | 2% | 20% | - | - | - | 4.01 | 1.01 | 4.19 | 0.84 | 1.74 | 0.90 | 80 |
|  | A10F5SU | - | - | 1% | 4% | 19% | 59% | 15% | 2% | 3.18 | 1.08 | 3.24 | 1.03 | 2.90 | 0.72 | 100 |
|  | A10F5HA2 | 89% | - | - | - | - | - | 3% | 8% | 3.89 | 0.99 | 3.39 | 1.08 | 4.09 | 0.72 | 109 |
|  | A10F5SA2 | - | 51% | 8% | 28% | 14% | - | - |  | 3.49 | 0.94 | 3.91 | 0.89 | 1.84 | 0.89 | 75 |
| **A11** | A11F12HA1 | 98% | - | - | - | - | 1% | - | 1% | 4.56 | 0.76 | 4.35 | 0.91 | 4.64 | 0.80 | 85 |
|  | A11F12SA | - | 54% | 17% | 12% | 8% | - | 1% | 9% | 3.53 | 1.07 | 3.61 | 0.79 | 2.26 | 1.03 | 125 |
|  | A11F12NE-SU | 4% | 1% | - | - | - | 37% | 52% | 6% | 3.02 | 1.15 | 2.70 | 1.12 | 3.22 | 0.61 | 81 |
|  | A11F12DI | 9% | 1% | 63% | 3% | - | 5% | 11% | 8% | 3.07 | 1.31 | 3.28 | 1.07 | 2.80 | 0.80 | 92 |
|  | A11F12SU | - | 4% | 5% | 9% | 2% | 31% | 21% | 28% | 2.80 | 1.21 | 2.87 | 1.01 | 2.96 | 0.63 | 108 |
|  | A11F12HA2 | 79% | 1% | - | - | - | - | - | - | 4.00 | 1.01 | 3.71 | 0.95 | 4.10 | 0.78 | 79 |
|  | A11F12AN | - | - | 24% | 73% | - | - | 3% |  | 3.86 | 1.12 | 4.02 | 0.96 | 2.16 | 1.05 | 63 |
| **A12** | A12F5AN | - | 14% | 22% | 41% | 5% | - | 1% | 18% | 3.11 | 1.08 | 3.28 | 0.93 | 2.27 | 0.72 | 95 |
|  | A12F5NE1 | - | 1% | 3% | - | 7% | 9% | 71% | 10% | 2.83 | 1.08 | 2.60 | 1.03 | 3.07 | 0.65 | 103 |
|  | A12F5HA | 74% | - | - | - | 1% | 6% | 14% | 4% | 3.35 | 1.16 | 3.05 | 1.05 | 3.93 | 0.68 | 92 |
|  | A12F5NE2 | - | 11% | - | - | 2% | 20% | 67% |  | 3.04 | 1.07 | 2.84 | 1.02 | 3.01 | 0.50 | 81 |
|  | A12F5DI-SA | - | 35% | 46% | 15% | 3% | - | 1% |  | 3.17 | 1.14 | 3.30 | 0.99 | 2.40 | 0.96 | 63 |
|  | A12F5SA | - | 66% | 17% | 14% | 3% | - | - |  | 3.73 | 1.09 | 4.05 | 0.86 | 1.96 | 0.84 | 56 |
|  | A12F5SA-AF | 2% | 42% | 2% | 9% | 35% | 11% | - |  | 3.04 | 1.14 | 3.48 | 1.09 | 2.00 | 0.66 | 52 |
| **A13** | A13M5NE1 | 26% | 1% | 1% | - | 1% | 3% | 56% | 13% | 2.80 | 1.10 | 2.61 | 0.92 | 3.34 | 0.65 | 114 |
|  | A13M5HA | 86% | - | - | - | - | 3% | 6% | 5% | 3.62 | 0.90 | 3.13 | 0.91 | 3.76 | 0.73 | 99 |
|  | A13M5NE2 | 1% | - | 2% | - | 2% | 1% | 92% | 2% | 3.23 | 1.05 | 2.82 | 0.97 | 3.25 | 0.54 | 101 |
|  | A13M5DI-AN | 1% | 18% | 33% | 31% | 1% | - | 2% | 14% | 3.30 | 1.15 | 3.40 | 0.93 | 2.28 | 0.92 | 131 |
|  | A13M5SA | - | 71% | 3% | 2% | 4% | - | 16% | 5% | 3.30 | 1.16 | 3.12 | 1.08 | 2.30 | 0.79 | 110 |
|  | A13M5DI | 4% | 7% | 55% | 22% | 1% | - | 11% |  | 2.87 | 1.02 | 3.31 | 1.04 | 2.34 | 0.87 | 70 |
| **A14** | A14M7HA-SU | 46% | 1% | - | - | - | 46% | 6% | 1% | 3.27 | 0.89 | 2.94 | 1.05 | 4.00 | 0.80 | 140 |
|  | A14M7DI-SA | - | 21% | 25% | 10% | 10% | 4% | 14% | 16% | 3.09 | 1.18 | 3.22 | 1.10 | 2.56 | 0.91 | 95 |
|  | A14M7NE | 2% | 20% | 1% | 1% | 11% | 4% | 52% | 9% | 2.88 | 1.13 | 2.78 | 1.13 | 2.87 | 0.68 | 90 |
|  | A14M7SA | - | 43% | 3% | 5% | 13% | 3% | 20% | 13% | 3.15 | 1.25 | 3.18 | 1.14 | 2.43 | 0.78 | 85 |
|  | A14M7HA | 68% | 1% | - | - | - | 19% | 7% | 5% | 3.23 | 1.02 | 2.95 | 1.04 | 3.60 | 0.79 | 80 |
| **A15** | A15M12SU | - | 5% | 4% | 17% | 10% | 53% | 4% | 8% | 3.25 | 1.08 | 3.45 | 0.94 | 2.77 | 0.70 | 129 |
|  | A15M12HA1 | 71% | 4% | - | 10% | 3% | 8% | 1% | 3% | 3.39 | 1.45 | 4.11 | 1.04 | 3.75 | 1.19 | 104 |
|  | A15M12HA2 | 77% | - | - | - | - | 13% | 7% | 2% | 3.41 | 0.97 | 3.23 | 0.97 | 3.86 | 0.59 | 95 |
|  | A15M12SA1 | - | 74% | 1% | 19% | 5% | - | - | 1% | 4.46 | 0.99 | 4.68 | 0.56 | 1.45 | 0.88 | 105 |
|  | A15M12SA2 | - | 77% | 3% | 9% | 9% | 1% | - | 1% | 4.05 | 1.00 | 4.17 | 0.77 | 1.69 | 0.65 | 99 |
|  | A15M12NE | - | 4% | 4% | 11% | 5% | 22% | 53% |  | 2.64 | 1.14 | 2.73 | 0.98 | 2.90 | 0.68 | 70 |
| **A16** | A16F6NE | 14% | 1% | 1% | - | 1% | 9% | 62% | 12% | 2.75 | 1.13 | 2.41 | 0.95 | 3.19 | 0.66 | 131 |
|  | A16F6HA | 72% | 1% | - | - | - | 13% | 8% | 6% | 3.23 | 1.06 | 3.00 | 1.03 | 3.86 | 0.72 | 108 |
|  | A16F6AF1 | - | 15% | 4% | 2% | 56% | 19% | - | 3% | 3.49 | 1.16 | 3.76 | 1.03 | 2.09 | 0.83 | 93 |
|  | A16F6SA1 | - | 69% | 2% | 16% | 8% | 1% | - | 4% | 3.45 | 1.08 | 3.48 | 0.82 | 2.09 | 0.74 | 92 |
|  | A16F6DI-AN | - | 12% | 44% | 29% | 1% | 1% | 12% |  | 3.19 | 1.17 | 3.39 | 0.97 | 2.53 | 0.83 | 70 |
|  | A16F6AF2 | - | 24% | 2% | 6% | 61% | 5% | 2% |  | 3.25 | 1.03 | 3.66 | 0.88 | 2.10 | 0.84 | 59 |
|  | A16F6SA2 | - | 65% | 1% | 31% | 3% | - | - |  | 3.97 | 1.15 | 4.29 | 0.82 | 1.80 | 1.06 | 66 |
| **A17** | A17F9HA1 | 98% | 1% | - | - | - | 1% | - | - | 4.46 | 0.82 | 4.22 | 0.77 | 4.51 | 0.74 | 127 |
|  | A17F9HA2 | 94% | 1% | - | - | - | 3% | 1% | 1% | 4.49 | 0.83 | 4.19 | 0.76 | 4.39 | 0.82 | 98 |
|  | A17F9DI | - | 7% | 40% | 21% | 2% | 1% | 3% | 27% | 3.21 | 1.08 | 3.42 | 0.95 | 2.49 | 0.78 | 110 |
|  | A17F9SA-SU-AF | - | 36% | 6% | 3% | 25% | 28% | 1% | 2% | 3.65 | 1.03 | 3.49 | 0.94 | 2.24 | 0.75 | 102 |
|  | A17F9NE | - | 3% | 5% | 11% | 2% | - | 53% | 26% | 3.14 | 1.10 | 3.12 | 0.99 | 3.09 | 0.74 | 130 |
|  | A17F9SU-AN | - | - | 7% | 21% | 2% | 34% | 12% | 24% | 3.06 | 0.98 | 3.16 | 0.88 | 2.79 | 0.63 | 95 |
|  | A17F9SA | - | 46% | 16% | 5% | 15% | 16% | 2% |  | 3.34 | 1.21 | 3.52 | 0.96 | 2.26 | 0.81 | 58 |
| **A18** | A18M7HA1 | 72% | 1% | 2% | 5% | 3% | 10% | - | 7% | 3.41 | 1.24 | 3.85 | 0.93 | 3.62 | 1.10 | 119 |
|  | A18M7HA2 | 93% | 1% | - | 1% | - | 2% | - | 3% | 4.01 | 0.97 | 3.85 | 0.82 | 4.23 | 0.60 | 101 |
|  | A18M7AF-DI | - | 8% | 34% | 1% | 39% | 10% | 3% | 6% | 3.22 | 1.11 | 3.49 | 0.89 | 2.34 | 0.86 | 111 |
|  | A18M7SA | - | 49% | 10% | 17% | 21% | - | 1% | 1% | 3.81 | 1.09 | 4.01 | 0.85 | 1.94 | 0.98 | 97 |
|  | A18M7SU1 | 10% | - | - | - | - | 86% | 3% | 1% | 4.00 | 1.11 | 4.01 | 0.92 | 3.75 | 0.73 | 95 |
|  | A18M7NE | - | 8% | 3% | 15% | 5% | 11% | 57% |  | 2.86 | 1.19 | 3.09 | 1.05 | 2.72 | 0.64 | 58 |
|  | A18M7DI | - | 13% | 51% | 1% | 33% | 2% | - |  | 3.46 | 1.08 | 3.65 | 0.96 | 2.14 | 0.91 | 78 |
|  | A18M7SU2 | - | 3% | - | - | 7% | 84% | 6% |  | 3.44 | 1.05 | 3.26 | 0.85 | 3.00 | 0.66 | 66 |
| **A19** | A19F6HA1 | 85% | - | - | - | - | 14% | - | 1% | 4.28 | 0.89 | 4.31 | 0.74 | 4.40 | 0.74 | 87 |
|  | A19F6HA2 | 58% | - | - | - | 1% | 1% | 26% | 15% | 3.46 | 0.99 | 3.07 | 1.04 | 3.84 | 0.67 | 114 |
|  | A19F6NE1 | - | 2% | - | - | 12% | 16% | 62% | 7% | 2.93 | 1.06 | 2.77 | 1.10 | 3.04 | 0.60 | 80 |
|  | A19F6SA | - | 78% | 4% | 11% | 6% | - | - | 1% | 4.15 | 1.02 | 4.21 | 0.90 | 2.05 | 1.11 | 138 |
|  | A19F6SU | 10% | - | - | 2% | - | 74% | 9% | 5% | 3.48 | 1.19 | 3.83 | 0.91 | 3.59 | 0.80 | 97 |
|  | A19F6AN | - | 23% | 5% | 60% | 11% | - | - | 2% | 3.95 | 1.08 | 4.37 | 0.76 | 1.82 | 0.98 | 83 |
|  | A19F6NE2 | - | 10% | 2% | 2% | 19% | 2% | 66% |  | 2.8 | 1.18 | 2.79 | 1 | 2.77 | 0.79 | 56 |
