# Supplementary material for: The Tromso Infant Faces Database (TIF): Development, Validation and Application to Assess Parenting Experience on Clarity and Intensity Ratings
Source: Front Psychol. 2017 Mar 24;8:409. doi: 10.3389/fpsyg.2017.00409 (PMC5364182; doi:10.3389/fpsyg.2017.00409)
Supplement: Supplementary file 2 [file Table_2.docx]

| Supplementary Table 2: Validation data for the 30 images from Study 2 across all respondents (minimum N=389) | | | | | | | | | | | |
| --- | --- | --- | --- | --- | --- | --- | --- | --- | --- | --- | --- |
|  |  |  |  |  |  |  |  |  |  |  |  |
| **Image** | **Emotion** | **Accuracy** | if < 50 %. highest % of other emotion | **Mean Clarity** | **SD Clarity** | **Mean Intensity** | **SD Intensity** | **Mean Valence** | **SD Valence** | **Mean Genuineness** | **SD Genuineness** |
| A04M6HA | Happy | 99% |  | 4.54 | 0.81 | 4.41 | 0.75 | 4.62 | 0.75 | 4.57 | 0.75 |
| A11F12HA1 | Happy | 98% |  | 4.65 | 0.73 | 4.46 | 0.74 | 4.68 | 0.66 | 4.43 | 0.98 |
| A17F9HA2 | Happy | 88% |  | 4.14 | 1.00 | 3.99 | 0.88 | 4.38 | 0.69 | 4.32 | 0.83 |
| A06M5HA2 | Happy | 99% |  | 4.39 | 0.84 | 3.96 | 0.91 | 4.38 | 0.71 | 4.49 | 0.71 |
| A02F10HA2 | Happy | 95% |  | 4.22 | 1.06 | 4.19 | 0.09 | 4.61 | 0.74 | 4.51 | 0.83 |
| A17F9HA1 | Happy | 95% |  | 4.55 | 0.81 | 4.42 | 0.75 | 4.61 | 0.72 | 4.51 | 0.88 |
| A06M5NE1 | Neutral | 94% |  | 3.49 | 1.14 | 3.04 | 1.11 | 3.15 | 0.55 | 4.06 | 0.90 |
| A02F10NE | Neutral | 61% |  | 2.83 | 1.15 | 2.82 | 1.00 | 2.83 | 0.66 | 3.94 | 0.91 |
| A10F5NE | Neutral | 83% |  | 3.31 | 1.17 | 2.95 | 1.08 | 3.32 | 0.67 | 4.02 | 0.91 |
| A09F9NE1 | Neutral | 50% |  | 2.79 | 1.14 | 2.83 | 1.03 | 2.59 | 0.71 | 3.85 | 1.04 |
| A13M5NE2 | Neutral | 85% |  | 3.40 | 1.13 | 2.95 | 1.04 | 3.33 | 0.64 | 4.05 | 0.89 |
| A12F5NE1 | Neutral | 64% |  | 2.94 | 1.16 | 2.85 | 1.05 | 2.99 | 0.56 | 3.93 | 0.92 |
| A03F7NE1 | Neutral | 60% |  | 2.87 | 1.16 | 2.90 | 1.03 | 3.04 | 0.65 | 3.97 | 0.93 |
| A19F6NE2 | Neutral | 18% | 49 % fear | 2.84 | 1.22 | 3.00 | 1.04 | 2.36 | 0.78 | 4.01 | 0.94 |
| A05F7SU | Surprise | 43% | 37 % fear | 3.02 | 1.15 | 3.24 | 0.98 | 2.67 | 0.75 | 4.08 | 0.91 |
| A18M7SU1 | Surprise | 88% |  | 3.82 | 1.18 | 4.05 | 0.97 | 3.82 | 0.84 | 4.26 | 0.93 |
| A08M12SU | Surprise | 45% | 41 % fear | 3.25 | 1.19 | 3.77 | 0.94 | 2.41 | 0.88 | 4.10 | 0.97 |
| A19F6SU | Surprise | 91% |  | 4.00 | 1.10 | 4.13 | 0.90 | 3.47 | 0.78 | 4.18 | 1.06 |
| A04M6SU | Surprise | 47% | 33 % fear | 3.16 | 1.18 | 3.50 | 0.93 | 2.53 | 0.82 | 4.05 | 0.93 |
| A15M12SU | Surprise | 54% |  | 3.28 | 1.22 | 3.56 | 1.00 | 2.69 | 0.82 | 4.19 | 0.87 |
| A18M7SU2 | Surprise | 36% | 28 % fear | 2.96 | 1.15 | 3.20 | 1.01 | 2.57 | 0.77 | 4.04 | 0.94 |
| A03F7SU | Surprise | 22% | 65 % neutral | 3.07 | 1.04 | 3.04 | 1.02 | 2.99 | 0.63 | 4.14 | 0.90 |
| A07M4SA | Sad | 76% |  | 3.86 | 1.11 | 4.16 | 0.85 | 1.90 | 1.03 | 4.38 | 0.94 |
| A09F9SA | Sad | 60% |  | 3.69 | 1.18 | 4.27 | 0.79 | 1.85 | 1.06 | 4.37 | 0.84 |
| A03F7SA | Sad | 45% | 29 % fear | 3.76 | 1.15 | 4.35 | 0.73 | 1.80 | 1.05 | 4.48 | 0.74 |
| A15M12SA2 | Sad | 60% |  | 3.63 | 1.14 | 4.00 | 0.86 | 1.98 | 0.98 | 4.23 | 0.93 |
| A10F5SA1 | Sad | 40% | 52 % fear | 3.77 | 1.05 | 4.16 | 0.84 | 1.76 | 0.93 | 4.40 | 0.77 |
| A16F6SA1 | Sad | 45% | 21 % disgust | 3.19 | 1.19 | 3.56 | 0.93 | 2.13 | 0.85 | 3.98 | 1.00 |
| A13M5SA | Sad | 71% |  | 3.42 | 1.22 | 3.34 | 1.15 | 2.14 | 0.84 | 4.23 | 0.89 |
| A19F6SA | Sad | 62% |  | 3.79 | 1.18 | 4.19 | 0.84 | 1.82 | 0.96 | 4.27 | 0.92 |
